# Critical Care Management of Severe Asthma Exacerbations

**DOI:** 10.3390/jcm13030859

**Published:** 2024-02-01

**Authors:** Shameek Gayen, Stephen Dachert, Bilal H. Lashari, Matthew Gordon, Parag Desai, Gerard J. Criner, Juan Carlos Cardet, Kartik Shenoy

**Affiliations:** 1 Department of Thoracic Medicine and Surgery, Lewis Katz School of Medicine at Temple University Hospital, Philadelphia, PA 19140, USA; stephen.dachert2@tuhs.temple.edu (S.D.); bilal.lashari@tuhs.temple.edu (B.H.L.); matthew.gordon@tuhs.temple.edu (M.G.); parag.desai@tuhs.temple.edu (P.D.); gerard.criner@tuhs.temple.edu (G.J.C.); kartik.shenoy@tuhs.temple.edu (K.S.); 2 Division of Allergy and Immunology, Department of Internal Medicine, University of South Florida, Morsani College of Medicine, Tampa, FL 33602, USA; jcardet@usf.edu

**Keywords:** asthma, bronchospasm, dynamic hyperinflation, mechanical ventilation, non-invasive ventilation, extracorporeal life support

## Abstract

Severe asthma exacerbations, including near-fatal asthma (NFA), have high morbidity and mortality. Mechanical ventilation of patients with severe asthma is difficult due to the complex pathophysiology resulting from severe bronchospasm and dynamic hyperinflation. Life-threatening complications of traditional ventilation strategies in asthma exacerbations include the development of systemic hypotension from hyperinflation, air trapping, and pneumothoraces. Optimizing pharmacologic techniques and ventilation strategies is crucial to treat the underlying bronchospasm. Despite optimal pharmacologic management and mechanical ventilation, the mortality rate of patients with severe asthma in intensive care units is 8%, suggesting a need for advanced non-pharmacologic therapies, including extracorporeal life support (ECLS). This review focuses on the pathophysiology of acute asthma exacerbations, ventilation management including non-invasive ventilation (NIV) and invasive mechanical ventilation (IMV), the pharmacologic management of acute asthma, and ECLS. This review also explores additional advanced non-pharmacologic techniques and monitoring tools for the safe and effective management of critically ill adult asthmatic patients.

## 1. Introduction

Despite advances in outpatient asthma management, patients frequently present with severe asthma exacerbations. Severe exacerbations can progress to respiratory failure, profound respiratory acidosis, and respiratory arrest requiring intubation and invasive mechanical ventilation (IMV), often termed near-fatal asthma (NFA) [1]. The goal of mechanical ventilation is to support the cardiopulmonary system while severe bronchoconstriction resolves. However, IMV can be challenging and requires a detailed understanding of the pathophysiology of acute asthma exacerbations. In this review, we discuss the pathophysiology and the optimal critical care management of severe asthma exacerbations, including pharmacologic, ventilatory, and utilization of extracorporeal life support techniques. We also discuss the role and limitations of various salvage techniques and monitoring entities, providing a comprehensive yet practical review of the management of critically ill adult asthmatic patients. 

## 2. Pathophysiology

The hallmark of asthma is airway hyperresponsiveness (AHR) leading to reversible airway obstruction. AHR is an exaggerated bronchoconstrictive response to a given stimulus and is associated with chronic inflammatory responses seen in most patients with asthma [2]. Chronic inflammation in asthma in most instances is driven by inappropriate responses from T-helper type (Th)-2 cells and innate lymphoid cells (ILC) to allergens and other stimuli with a resulting downstream cascade of type 2 inflammatory mediators. Repeated activation of these pathways results in airway remodelling [3]. Structural changes seen in airway remodelling include subendothelial thickening, smooth muscle hypertrophy and hyperplasia, matrix deposition, and vascular changes, which lead to airway thickening, narrowing, and a decrease in airway compliance [4]. In acute exacerbations, environmental triggers or respiratory infections activate inflammatory cascades resulting in bronchospasm, airway obstruction, dynamic hyperinflation, and ultimately respiratory failure and cardiopulmonary arrest [1].

### 2.1. Bronchoconstriction 

In asthma, the propensity for contraction of airway smooth muscle (ASM) leading to bronchoconstriction can be secondary to intrinsic and extrinsic factors. Intrinsic factors include alterations in ASM gene expression in certain cohorts of patients with asthma [5]. Extrinsic factors lead to the activation of pro-inflammatory cytokines, including IL-4, IL-13, and tumour necrosis factor-alpha (TNF-α), which have been shown in vitro to affect airway smooth muscle contractility [6]. 

### 2.2. Dynamic Hyperinflation 

**Figure 1:**
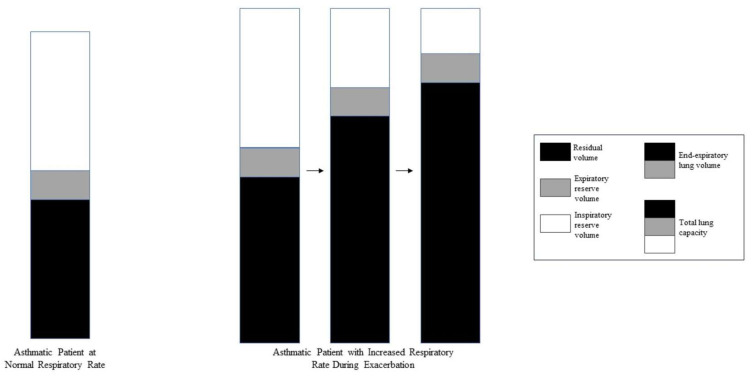
Air trapping and dynamic hyperinflation during asthma exacerbations. During acute asthma exacerbations, air trapping and dynamic hyperinflation occur as the respiratory rate increases due to reduced exhalation time. Residual volume and end-expiratory volume increase while inspiratory reserve volume decreases.

**Figure 2:**
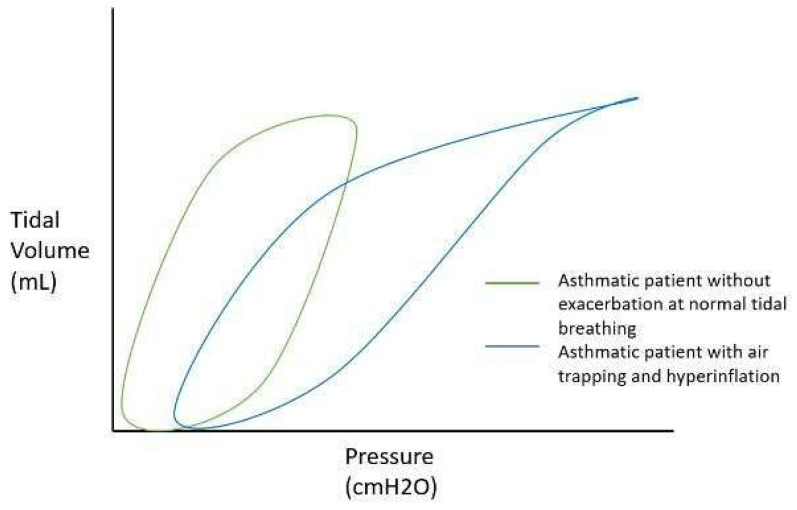
Effect of air trapping and hyperinflation on the pressure–volume curve in status asthmaticus. A well-controlled asthmatic patient without exacerbation will have a normal-appearing pressure–volume loop (green curve). In status asthmaticus, bronchoconstriction, dynamic hyperinflation, and air trapping occur, leading to decreased lung compliance and the requirement of higher peak pressures in order to reach the desired tidal volume, resulting in a “bird beaking” pattern. Additionally, the curve is shifted to the right, representing intrinsic positive end-expiratory pressure (PEEP) secondary to air trapping (blue curve).

**Figure 3:**
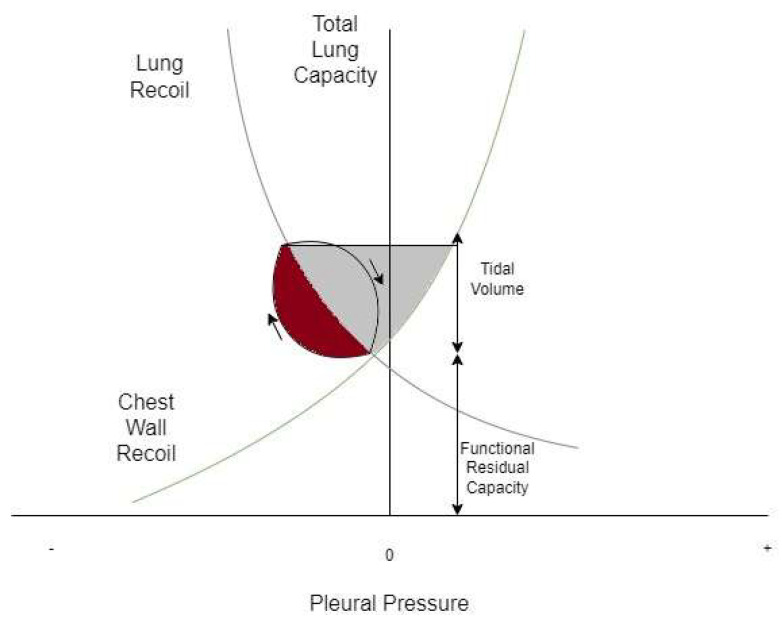
Campbell diagram of the normal lung at tidal breathing. Lung recoil is plotted against chest recoil. The loop demonstrates one breath from functional residual capacity in the direction of the arrows. The red shaded area demonstrates the work done against resistance and the grey shaded area demonstrates the work done against the elastic recoil of the lung and chest wall.

**Figure 4:**
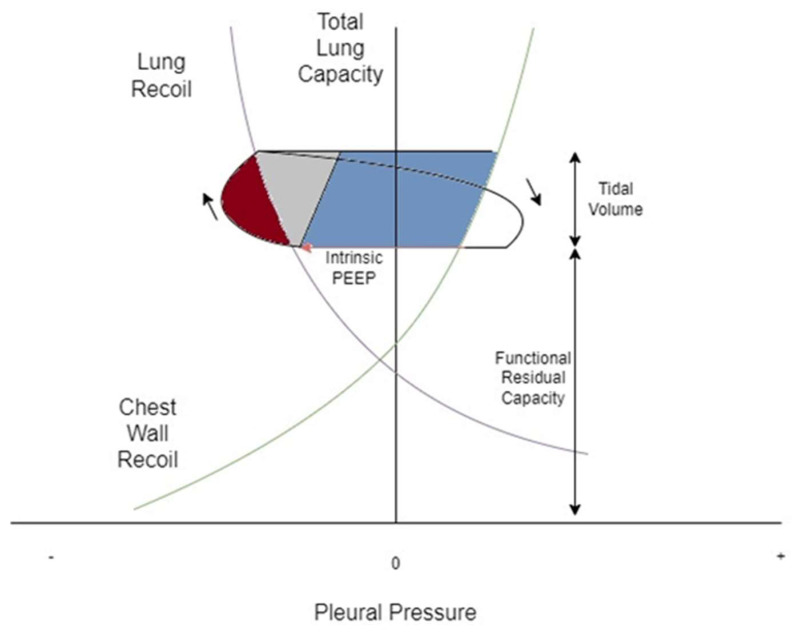
Campbell diagram of the lung of a subject in severe asthma exacerbation. Lung recoil is plotted against chest recoil. The loop demonstrates one breath from functional residual capacity in the direction of the arrows, here occurring at a higher volume. The red arrow represents intrinsic PEEP. The red shaded area demonstrates the work done against resistance and the grey shaded area demonstrates the work done against elastic recoil of lung and chest wall. Here, the blue shaded area represents the work done to overcome intrinsic PEEP.

Dynamic hyperinflation during acute bronchospasm is thought to be secondary to expiratory flow limitation resulting in compromised alveolar emptying and air trapping during tidal breathing [7]. This results in increased end-expiratory lung volume (EELV), measured as auto-positive end-expiratory pressure (PEEP) during IMV, and decreased inspiratory capacity (IC) (Figure 1). When end-inspiratory lung volume (EILV) approaches total lung capacity (TLC), the inspiratory reserve volume (IRV) decreases, and the ratios of tidal volume to IC (Vt/IC) and EILV/VT increase, causing tidal ventilation to occur close to TLC. Each breath is on the stiffer, less compliant portion of the pressure–volume curve (Figure 2, Figure 3 and Figure 4), putting additional stress on respiratory muscle fibres for a given Vt, causing respiratory muscle fatigue and dyspnea [8]. Additionally, dynamic hyperinflation causing large lung volumes can have profound cardiac effects due to decreased blood flow in thoracic vessels. This can lead to decreased left ventricular preload and afterload, decreased cardiac output, increased pulmonary vascular resistance, and even the development of pulsus paradoxus in unassisted breathing [1].

### 2.3. Mucus Plugging 

Mucus hypersecretion occurs in chronic and acute asthma exacerbations due to increased airway wall epithelial goblet cells. IL-4 and IL-13 have also been shown to play roles in mucus hypersecretion [9]. Autopsy studies of asthmatic patients have demonstrated varying degrees of mucus plugging, with the highest mucus burden seen in fatal asthma [10]. Patients with a higher burden of mucus plugging seen on chest CT during exacerbation have been shown to have worsening FEV1 at the time of exacerbation and decreased responsiveness to corticosteroids and inhaled bronchodilators [11]. 

## 3. Standard Pharmacologic Management

### 3.1. Treating Bronchospasm 

Common bronchodilator therapies are β-2-adrenergic agonists and anticholinergics. Short-acting β-2-adrenergic agonists (SABA) such as albuterol have a rapid onset of action and provide significantly more bronchodilation than anticholinergics [1]. Repetitive or continuous SABA administration is considered the cornerstone of acute asthma exacerbation therapy. Continuous SABA nebulization may allow for more consistent delivery of medication and deeper tissue penetration, resulting in enhanced bronchodilation, and may be more effective in severe airflow obstruction [12]. While continuous SABA nebulization has been found to be more efficient and effective than intermittent SABA nebulization in treating children with status asthmaticus or severe asthma, to our knowledge such benefits have not been found in adults; similar safety, morbidity, and mortality have been found between continuous and intermittent SABA nebulization in adults [13,14,15]. 

Bronchodilatory response to albuterol can vary among patients, in which case a dose–response titration of nebulized albuterol could be beneficial [16]. It is important to note that high-dose β-2-agonist therapy can cause lactic acidosis via increased pyruvate production from β-2-adrenergic stimulation of glycolysis and lipolysis [17]. This is a type B lactic acidosis that is not indicative of tissue hypoperfusion, though it is important to rule this out before attributing lactic and metabolic acidosis to β-2-agonism [17]. Intravenous β-agonists may be considered if the response to nebulized albuterol remains poor. However, their use has not demonstrated improved efficacy as compared to inhaled β-agonists and may have more severe side effects, including hypokalaemia, myocardial ischemia, and other cardiovascular events [1,12].

Anticholinergics counteract the effects of acetylcholine on muscarinic receptors, promoting bronchodilation via smooth muscle relaxation and inhibition of mucus secretion [18]. Ipratropium bromide is the most commonly used anticholinergic for acute asthma exacerbations, but its onset of action is 60–90 min to peak [19]. In adults with moderate-to-severe asthma exacerbations, the use of ipratropium with SABA in the emergency room is recommended and associated with fewer hospitalizations [20]. To our knowledge, there are no recommendations or evidence supporting the use of ipratropium in adult patients hospitalized with severe asthma exacerbations. 

Intravenous magnesium sulphate is a second-line bronchodilator that causes smooth muscle relaxation and bronchodilation via calcium channel blockage [21]. Intravenous magnesium at a dose of 2 g can be beneficial in adults with acute severe asthma, including improving pulmonary function, with minimal side effects [22,23,24,25]. However, it is important to note that a recent study showed that in children with moderate-to-severe asthma exacerbations, intravenous magnesium was associated with increased exacerbation severity, increased risk for hospitalization, and no acceleration in exacerbation resolution among hospitalized patients [26]. 

### 3.2. Treating Airway Inflammation 

Systemic corticosteroid therapy is essential for the resolution of asthma exacerbations that are refractory to intensive bronchodilator therapy because persistent airflow obstruction is likely due to airway inflammation [27] Optimal corticosteroid dosing in severe asthma exacerbations is unclear. While the equivalent of prednisone 40 mg to 60 mg daily is standard for acute asthma exacerbations, higher doses (60–80 mg every 6 to 8 h) are often given in severe exacerbations [1]. Oral and intravenous administration are equally efficacious [12]. 

Leukotriene receptor antagonists (LRAs) function as both bronchodilators and anti-inflammatory agents, though less potent than SABA and corticosteroids, respectively [12]. Leukotrienes are increased in asthma exacerbations and cause bronchoconstriction, mucus hypersecretion, and inflammatory cell recruitment; antagonizing their action could be effective in this setting [28]. Intravenous LRAs, such as montelukast, have a rapid and significant improvement in pulmonary function and can act as an adjunct therapy to prevent intubation in this patient population [12]. 

Anti-interleukin-5 agents such as benralizumab and mepolizumab, currently approved to prevent asthma exacerbations in eosinophilic asthma, have been found in case reports to improve symptoms of NFA and reduce the need for IMV [29,30]. The use of benralizumab in acute asthma in the emergency department has been shown to significantly reduce blood eosinophil count and the rate and severity of subsequent exacerbations; the effect on symptom improvement during the presenting exacerbation was not studied [31]. Further trials are needed to evaluate the efficacy of these agents in acute asthma exacerbations. 

## 4. Non-Invasive Ventilation

Non-invasive ventilation (NIV) has been shown to reduce IMV rates and mortality in respiratory failure related to acute exacerbations of chronic obstructive pulmonary disease (COPD) [32]. Despite similar pathophysiology of obstruction and dynamic hyperinflation experienced during acute asthma exacerbations, similarly robust evidence for the use of NIV in acute asthma exacerbations does not exist. Only a few randomized clinical trials have attempted to investigate the potential benefits of NIV in acute asthma exacerbations (Table 1). Despite some benefits, these trials were not powered to assess for prevention of IMV or change in mortality. As a result, the European Respiratory Society and American Thoracic Society do not include a recommendation for a trial of NIV during an exacerbation of asthma [33].

Despite limited evidence, the use of NIV in acute asthma has increased from 18.5% in 2010 to 29.9% in 2017. More recently, a multicentre retrospective cohort study of 53,654 patients found that using NIV in acute asthma was associated with lower odds of receiving IMV and mortality [41]. 

### 4.1. Mechanical Effects on Bronchial Dilation, FEV1, and PEFR 

Continuous positive airway pressure (CPAP) has been shown in patients with asthma exacerbations to increase the diameter of small and medium bronchi [42]. Initiation of NIV at a level of inspiratory positive airway pressure (IPAP) of 8 cm H_2_O and expiratory positive airway pressure (EPAP) of 6 cm H_2_O leads to a significantly increased mean per cent change in FEV1 compared to a lower pressure group and a non-NIV group after 20, 40, and 60 min [38]. The use of bi-level positive airway pressure (BIPAP) along with multiple treatments with aerosolized bronchodilators increased the peak expiratory flow rate (PEFR) compared to non-BiPAP controls [34]. 

### 4.2. Offloading of Muscles of Respiration

Patients with mild to moderately severe asthma exacerbations who were randomized to receive BIPAP had improvement in the BORG scale of perceived exertion and significantly lower work of breathing as measured by accessory muscle compared to controls [38]. This indicates that offloading respiratory muscles and preventing respiratory muscle fatigue are benefits of NIV in this patient population. 

### 4.3. Assessing for Non-Invasive Ventilation Use

There are no widely accepted practical indications for the initiation of NIV during acute asthma. Suggested criteria and contraindications are shown in Table 2. NIV initiation should be carried out in an ICU setting, where IMV can occur quickly if there is a failure of NIV [43]. 

### 4.4. Complications of Non-Invasive Ventilation 

Potential adverse effects of NIV include barotrauma, such as pneumothorax, related to positive pressure. The potential for pneumonia related to aspiration events surrounding NIV administration also remains a concern. There is an increased incidence of pneumonia and severe sepsis in patients who failed NIV and required IMV [41]. 

## 5. Mechanical Ventilation in Severe Acute Asthma Exacerbations 

### 5.1. Indications for Mechanical Ventilation 

The primary indications for IMV in severe asthma exacerbations, or status asthmaticus, are respiratory arrest, extreme respiratory muscle fatigue, and encephalopathy [44]. This often manifests as worsening respiratory acidosis and/or progressive respiratory fatigue. If NIV fails, intubation and IMV are required. At this point, it is important to assess pulmonary hyperinflation. 

### 5.2. Assessing Pulmonary Hyperinflation 

Assessment of hyperinflation during IMV can be performed by measuring plateau pressure (P_plat_) and auto-PEEP during volume control ventilation [44]. In non-obese patients with asthma, compliance is typically normal, so P_plat_ is primarily influenced by hyperinflation. P_plat_ can be measured during an inspiratory hold manoeuvre on the ventilator, while auto-PEEP can be measured during an expiratory hold manoeuvre. Sedation and ventilator synchrony is necessary to perform an expiratory hold manoeuvre and will help minimize auto-PEEP by removing additional patient-triggered breaths. While it is unclear at which levels of P_plat_ and auto-PEEP there is an increased risk of complications, a suggested P_plat_ target is < 30 cm H_2_O, while auto-PEEP can be 10 cm H_2_O or higher [44]. 

### 5.3. Ventilator Settings and Strategies 

**Figure 5:**
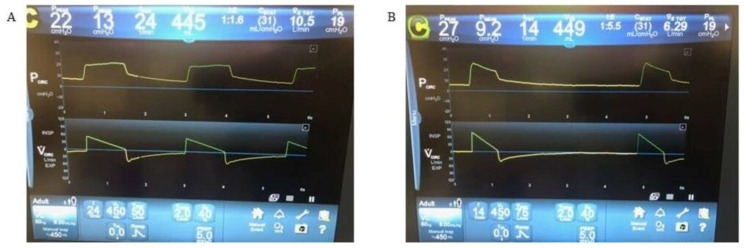
Inspiratory to expiratory time during mechanical ventilation in acute asthma. (**A**) Mechanical ventilation with respiratory rate set to 24 breaths per minute in patients with acute asthma. The I:E ratio is close to 1:1, and the volume waveform does not quite reach the baseline before the next breath starts. This will lead to air trapping, auto-PEEP, and dynamic hyperinflation. (**B**) The same patient but with respiratory rate set to 14 breaths per minute. The I:E ratio is ~1:5, and the volume waveform clearly returns to baseline before the next breath is initiated; this will prevent air trapping, auto-PEEP, and dynamic hyperinflation. I:E: inspiratory to expiratory. PEEP: positive end-expiratory pressure.

Either pressure control or volume control ventilation can be utilized. The use of pressure control ventilation allows for better control over alveolar pressure but at the expense of controlling tidal volume and minute ventilation. The use of volume control ventilation allows for control of tidal volume but at the expense of controlling alveolar pressure. Regardless, ventilator settings should set low minute ventilation in order to allow for sufficient exhalation and prolongation of the inspiratory to expiratory (I:E) time ratio, which in turn leads to lower alveolar pressure. The optimal I:E time is 1:4 to 1:5 [45]. Decreasing respiratory rate is most effective at prolonging I:E time, and the target respiratory rate should be approximately 12–14 breaths/minute (Figure 5). Prolonging exhalation will reduce air trapping and subsequently decrease EELV, auto-PEEP, alveolar pressure, and dynamic hyperinflation. 

Other ventilator strategies include decreasing tidal volume and shortening inspiratory time (Table 3). Inspiratory time can be decreased either by increasing inspiratory flow in volume control ventilation (up to 80–100 L/minute) or by decreasing inspiratory time in pressure control ventilation. Specifically shortening inspiratory time will increase recorded peak inspiratory pressure (PIP) without increasing the risk of barotrauma, as this increased pressure does not reflect additional alveolar distension or increased intrathoracic pressure. 

### 5.4. Extrinsic PEEP Delivery 

The development of auto-PEEP in patients with airflow obstruction actively breathing leads to increased work of breathing, as patients need to generate additional negative intrathoracic pressure to overcome their auto-PEEP. Offsetting auto-PEEP with extrinsic PEEP to reduce this effect is the rationale for using NIV in acute exacerbations of COPD and, in some cases, asthma [43]. However, there is limited evidence to support the benefit of extrinsic PEEP delivery to patients requiring IMV for severe asthma exacerbations, where spontaneous breathing is minimal or absent, and it may, in fact, be harmful (Table 3). 

### 5.5. Complications of Mechanical Ventilation 

Prolonging expiration and the I:E time is necessary to minimize auto-PEEP in severe airflow obstruction. Large amounts of auto-PEEP lead to increased intrathoracic pressure, which is reflected by elevated pulmonary plateau and peak pressures and can result in hypotension and barotrauma [50]. 

The risk of hypotension is related to the degree of air trapping and hyperinflation, which causes decreased venous return and increased right ventricular (RV) afterload [44]. Additionally, severe hyperinflation due to insufficient exhalation set by the ventilator can cause decreased LV preload and afterload and decreased cardiac output [1]. When hypotension occurs in a mechanically ventilated patient with severe airflow obstruction, a 30 to 60 s apnoea trial can be performed, in addition to optimizing I:E time as described above [51]. If these interventions and intravenous fluid resuscitation do not improve hypotension, other causes, such as pneumothorax and decreased cardiac function, should be considered. 

The incidence of pneumothorax in patients treated with mechanical ventilation for severe asthma ranges from 3% to 6% [44]. Pneumothorax risk increases with hyperinflation, with a higher incidence seen when EILV exceeds 20 mL/kg [52]. Chest radiographs and bedside lung ultrasound can help diagnose pneumothorax, and chest tube placement via blunt dissection is preferred to avoid piercing a hyperinflated lung [51]. 

## 6. Additional Supportive Techniques 

### 6.1. Bronchoscopy 

Mucus plugging causes airway inflammation and obstruction and has been seen in fatal cases of asthma exacerbations [53]. Bronchoscopy in asthmatic patients on mechanical ventilation has been found to be effective in improving PaCO_2_ levels, airway compliance, and expediting extubation in case reports [54]. Although large studies evaluating bronchoscopy in severe asthma exacerbations are lacking, bronchoscopy can be considered when bronchospasm is not improving despite conventional therapy or if atelectasis and/or increasing peak airway pressures are seen. 

### 6.2. Sedation and Analgesia

Adequate sedation and analgesics are necessary to ensure ventilator synchrony, especially when the patient is difficult to ventilate. Propofol is a useful first choice as it allows for deep sedation. Propofol does not supply analgesia, and thus opioid medications are often used as adjunct pain relief. In cases where less deep sedation is needed, dexmedetomidine may be utilized. Dexmedetomidine is an α2 receptor agonist and has anxiolytic properties [1,12]. 

Ketamine is a sedative agent that has bronchodilator properties as well. Ketamine has several mechanisms of action that promote bronchodilation, including N-methyl-D-aspartic acid antagonism, reduction in nitric oxide levels, reversal of histamine-induced bronchoconstriction, increase in synaptic catecholamine levels, and reduction in airway smooth muscle spasm via vagal effects [55,56]. The use of ketamine as a continuous infusion in patients receiving IMV for severe bronchospasm leads to reduced PIP, improved gas exchange, and successful weaning of IMV [55]. However, the use of ketamine in this setting is not universally recommended as only a few trials with large heterogeneity in design and outcomes have been published; further clinical trials are warranted to establish the role of ketamine in severe asthma exacerbations [57]. 

### 6.3. Paralysis 

The main objective of sedation and paralysis in asthmatic patients is to ensure the prevention of patient–ventilator dyssynchrony. Respiratory failure resulting from status asthmaticus may lead to vigorous respiratory effort, which may predispose to patient self-inflicted lung injury. When considered in the context of IMV, the effect of barotrauma and volutrauma may become compounded. While therapeutic principles dictate low tidal volume ventilation with a prolonged I:E time, these are non-physiologic and poorly tolerated in the awake patient. The use of neuromuscular blockers may be obligatory, especially at the initiation of IMV. Rocuronium, vecuronium, and cisatracurium are commonly used in the United States (US) and may be used barring specific contraindications. Continuous infusion of neuromuscular blockers is best reserved for the small subset of patients that require frequent intermittent boluses, despite adequate analgosedation. Prolonged ongoing neuromuscular blockade carries a small but significant risk of severe critical illness myopathy, especially with concomitant corticosteroid use [58,59]. 

### 6.4. Obligatory Permissive Hypercapnia 

Hypercapnia during mechanical ventilation of the patient with status asthmaticus is common and secondary to two distinct mechanisms. The first is physiologic and the result of alveolar hyperdistension leading to an increased physiologic dead space. The second is iatrogenic and occurs when low minute ventilation is used to prevent dynamic hyperinflation and its deleterious effects on cardiac output. It is interesting to note that efforts to mitigate the former by increasing minute ventilation will lead to dynamic hyperinflation and progressive increase in alveolar dead space, thereby forcing the latter’s acceptance. Therefore, hypercapnia is best understood as “obligatory” and should be tolerated to allow enough time for acute bronchospasm to resolve. Serious adverse effects of hypercapnia are uncommon in severe asthma, especially cerebral oedema [60,61]. 

A safe PaCO_2_ level is difficult to determine and requires an astute understanding of the patient. CO_2_ itself is remarkably well tolerated, especially when chronic. However, an acute rise in CO_2_ in the form of respiratory acidosis may lead to cardiac arrhythmias, hyperkalaemia, or hemodynamic instability. In the absence of these, correction of acidosis may not be necessary. Close monitoring and attention to chemistry, telemetry, and surrogates of cardiac output such as urine output are invaluable in this situation. 

### 6.5. Acidemia and the Use of Buffer Solutions 

A lower limit of pH in the critically ill asthmatic patient has not been established. However, the acute respiratory distress syndrome literature reports pH as low as 7.20 being well tolerated; it is reasonable to assume the same to be true in severe asthma [62,63]. 

Electrolyte imbalance, hemodynamic instability, and cardiac arrhythmias are likely at pH below 7.20, and the use of buffer solutions may become necessary. The only buffer solution readily available in the USA for clinical use is sodium bicarbonate, which, while temporizing pH acutely, dissociates into H^+^ and HCO_3_^−^. HCO_3_ will be converted to H_2_O and CO_2_ and increase hypercapnia, which can be troublesome if acute bronchospasm has not been relieved. Since most patients have some degree of improvement in ventilation in the first 12–24 h of critical care, using buffers may be useful. Using intermittent sodium bicarbonate boluses, instead of a continuous infusion, will decrease the likelihood of hypervolemia and the need for diuretic therapy. Tromethamine (THAM) is a buffer solution within the pH range of 7–9 that does not dissociate to CO_2_ when administered. THAM is not readily available in the USA, and its clinical use is limited. The use of bicarbonate and THAM will result in post-hypercapnic metabolic alkalosis that is difficult to correct; this may be more severe with THAM administration [64]. 

### 6.6. Heliox 

Helium, being less dense than air, offers a physical advantage when mixed with oxygen in acute bronchospasm. When mixed with oxygen in a 70:30 ratio, the mixture has a viscosity similar to air but with considerably lower density. This attribute becomes important by lowering the Reynolds number, which promotes laminar flow. Laminar flow in turn decreases resistance and allows gas to pass through bronchospastic airways. The use of Heliox may decrease auto-PEEP by providing this laminar flow in patients with airway disease [65]. 

It is important to note that since Heliox is usually bled through the ventilator circuit, it adds volume to that delivered by the ventilator thereby increasing the PIP display. The Vt displayed by the ventilator is usually erroneous. 

### 6.7. Use of Inhaled Anaesthetic Agents 

Inhaled anaesthetic agents have been part of the classical teaching for severe asthma owing to the bronchodilatory properties of the fluorinated ethers. While halothane, isoflurane, and sevoflurane have bronchodilatory properties, the use of isoflurane is the only one that has demonstrated benefit in reducing airway resistance and auto-PEEP in a small clinical study [66]. 

The institution of inhaled anaesthetics is challenging and requires familiarity with anaesthetics and delivery systems. Depending on available resources, the use of an operating room (OR) ventilation system may be required, as well as portable gas tanks. Familiarity with the limitations associated with inhaled anaesthetics is essential. During the delivery of inhaled anaesthetics, there is an inability to nebulize and actively humidify inspired gas. Certain ventilators may be unable to achieve inspiratory pressures to overcome airway pressures in severe acute asthma and would not be able to deliver effective tidal volumes. Intimate knowledge and familiarity with available mechanical ventilators in the ICU and the OR is a prerequisite that must be fulfilled prior to consideration of using these agents [67]. 

## 7. Venovenous ECMO for Severe Asthma Exacerbations 

### 7.1. Indications 

Despite appropriate mechanical ventilation and pharmacologic therapy, some patients with severe asthma exacerbations still worsen. The use of venovenous extracorporeal membrane oxygenation (VV-ECMO) in these patients can be lifesaving. 

Indications for extracorporeal life support (ECLS) such as VV-ECMO include carbon dioxide (CO_2_) retention on IMV despite high plateau pressure and CO_2_ retention syndromes, including asthma exacerbations [68]. In severe asthma exacerbations, bronchospasm and airflow obstruction lead to elevated PIP and ineffective ventilation. In these cases, attempts at effective IMV can result in barotrauma and hemodynamic instability [44,46,69]. The early use of VV-ECMO in severe asthma exacerbations allows for the minimization of these adverse effects [45]. 

Hypercapnia is often the primary respiratory abnormality in severe asthma exacerbations. As such, the use of extracorporeal CO_2_ removal (ECCO_2_R) can be considered, with CO_2_ cleared from the blood via the ECMO circuit without oxygenating blood that travels through the circuit. In addition to case reports, there have been small studies examining ECCO_2_R for hypercapnia in asthma exacerbations. The rationale for its use was to improve hypercapnia and minimize dynamic hyperinflation; improvement in PaCO_2_ and the need for intubation were seen [70,71,72,73,74]. Although large studies are absent, ECCO_2_R can be effective in improving hypercapnia and minimizing dynamic hyperinflation. 

### 7.2. Management of Venovenous ECMO 

Initiation of VV-ECMO allows for lung protective ventilation, often termed “rest ventilator settings.” Typical “rest ventilator settings” allow extremely low minute ventilation and minimizing of hyperinflation [74]. While lung rest occurs, VV-ECMO allows for CO_2_ removal and oxygenation via the circuit membrane. CO_2_ removal is a function of ECMO sweep gas flow, which is typically initially set to 50–80 mL/kg/min and titrated to systemic pH and PaCO_2_ [75]. While neurologic injury from a fall in cerebral tissue oxygenation can occur due to rapid CO_2_ removal, this is more likely to occur in chronic lung disease with hypercapnia as opposed to asthma [76]. As underlying bronchospasm is treated, ventilator settings can be optimized from “rest settings” and VV-ECMO settings can be titrated down until ECMO decannulation is feasible. 

### 7.3. Outcomes of VV-ECMO for Severe Asthma 

Analyses of the international Extracorporeal Life Support Organization (ELSO) registry data found a survival rate greater than 83% in patients with severe asthma requiring VV-ECMO, with greater survival than other indications for ECMO and improvement in PIP and mean airway pressure. However, 28.3% of patients experienced haemorrhagic complications [50,77]. These studies highlight the effectiveness and high survival rate of VV-ECMO as a treatment modality for severe asthma exacerbations, though potential complications should be kept in mind. Haemorrhagic complications may be mitigated by the use of newer intravenous anticoagulants such as bivalirudin, a direct thrombin inhibitor, and nafamostat mesilate, a synthetic serine protease inhibitor; these have been shown to be effective in preventing thrombosis while reducing the risk of bleeding in patients maintained on ECMO [78,79].

## 8. Monitoring the Critically Ill Asthmatic Patient

There are several monitoring entities that can be utilized to ensure the effective and safe management of critically ill asthmatic patients, particularly those treated with invasive mechanical ventilation (Table 4).

## 9. Discussion and Conclusions

Severe asthma exacerbations are characterized by a substantial worsening in airway hyperresponsiveness, bronchoconstriction, and dynamic hyperinflation from a patient’s baseline. Understanding and managing these aspects is crucial in the optimal critical care management of the severe asthmatic patient. While pharmacotherapy addresses the underlying bronchoconstriction and airway inflammation, severe asthma exacerbations will often require IMV. Understanding the underlying physiology and lung mechanics in acute asthma exacerbations is important, as excessive ventilation can lead to hypotension and barotrauma; as such, controlled hypoventilation and I:E prolongation is essential. 

Despite optimal pharmacotherapy and IMV, patients can still worsen and require additional supportive measures. Such measures include ECLS, paralysis, Heliox, buffer solutions, and inhaled anaesthetic agents. An appropriate understanding of the role and limitations of these techniques is vital for their effective use. We have also highlighted several monitoring entities that can be used to help ensure effective and safe management of the critically ill asthmatic patient, a task that requires a deep understanding of the underlying physiology and the many targeted interventions available.

## 10. Future Directions

The optimal and universally accepted critical care management of severe asthma exacerbations is based on a handful of principles. However, there are several areas of management that warrant further evaluation via prospective randomized trials, namely the use of NIV, given the findings of a recent, large multicentre study, and the use of ECCO_2_R to improve hypercapnia and dynamic hyperinflation.

## Figures and Tables

**Table 1:** Randomized controlled trials investigating non-invasive ventilation in acute asthma.

Study	Number of Patients	Outcome
Pollack et al., 1995 [34]	40 patients in conventional nebulizer group, 60 patients in BiPAP group	ED management with NIV to deliver bronchodilators resulted in statistically significant improvement to peak expiratory flow rate (PEFR)
Holley et al., 2001 [35]	19 patients in NIV group, 16 in standard of care group	Trend toward decreased need for mechanical ventilation and decreased length of hospital stay in NIV group.
Soroksky et al., 2003 [36]	15 patients in NIV and conventional therapy group, 15 patients in conventional-therapy-only group	In patients with severe acute asthma, NIV showed statistically significant improvement to FEV1, and a reduction in need for hospitalization.
Gupta et al., 2010 [37]	28 patients in NIV and conventional therapy, 25 patients in conventional-therapy-only group	More patients receiving NIV experienced >50% improvement in FEV1 at 1, 2, 4 h compared to conventional therapy group, although not statistically significant. Significantly shorter ICU stay in NIV group.
Soma et al., 2008 [38]	16 patients with IPAP 8 cm H_2_O and EPAP 6 cm H_2_O, 14 patients with IPAP 6 cm H_2_O and EPAP 4 cm H_2_O), and 14 patients in conventional-therapy-only group	Statistically significant improvement to FEV1 in high-pressure group only compared to conventional therapy group. Significant improvement to dyspnoea as measured by Borg scale in high- and low-pressure groups compared to conventional therapy group.
Brandao et al., 2009 [39]	24 patients in NIV group (12 IPAP 15 cm H_2_O EPAP 5 cm H_2_O and 12 IPAP 15 cm H_2_O EPAP 10 cm H_2_O)	Statistically significant improvement to PEFR and FVC in high-EPAP group. Statistically significant improvement in PEFR and respiratory rate in the low-EPAP group compared to conventional therapy group.
Galindo-Filhr et al., 2012 [40]	11 patients received NIV and conventional therapy and 10 patients received conventional therapy	Statistically significant improvement in respiratory rate, FEV1, FVC, PEFR and IC in NIV group compared to conventional therapy group.

BiPAP: bi-level positive airway pressure; ED: emergency department; EPAP: expiratory positive airway pressure; FEV1: forced expiratory volume in 1 s; FVC: functional vital capacity; IC: inspiratory capacity; ICU: intensive care unit; IPAP: inspiratory positive airway pressure; NIV: non-invasive ventilation; PEFR: peak expiratory flow rate.

**Table 2:** Indications and contraindications for NIV initiation in acute asthma exacerbations.

**Indications**
Respiratory Rate > 25 breaths per minute
Heart Rate > 110 beats per minute
Accessory Respiratory Muscle Use
Hypoxemia with P/F ratio > 200
Hypercapnia with partial pressure carbon dioxide (PaCO_2_) < 60 mm Hg
FEV1 < 50% predicted following ≥ 2 consecutive nebulized bronchodilators
**Absolute Contraindications**
Decreased alertness
High risk for aspiration
Circumstances precluding proper mask seal
Physical restraints due to agitation
Lack of properly trained staff
**Relative Contraindications**
Hemodynamic instability
Severe hypoxemia (P/F ratio < 200)
Severe hypercapnia (PaCO_2_ > 60 mm Hg)

FEV1: forced expiratory volume in 1 s; P/F: partial pressure of oxygen to fraction of inspired oxygen ratio; PaCO_2_: partial pressure of carbon dioxide.

**Table 3:** Ventilator strategies in severe asthma exacerbations.

Ventilator Strategy	Effect
Controlled hypoventilation with low respiratory rate [46]	Hypercapnia without deleterious effects; no pneumothorax or mortality
Lower vs. higher minute ventilation— 10 L/min vs. 16 L/min vs. 26 L/min [47]	Increase in pulmonary hyperinflation, risk of hypotension and barotrauma at higher minute ventilation
Increased inspiratory flow and square waveform [47,48]	Reduced inspiratory time, prolonged I:E ratio but with minimal expiratory prolongation; minimal impact on hyperinflation at lower minute ventilation
Extrinsic PEEP application [49]	Applied PEEP worsened hyperinflation, hypotension without decreasing auto-PEEP

I:E: inspiratory:expiratory; PEEP: Positive end-expiratory pressure.

**Table 4:** Monitoring entities and their usefulness in severe asthma.

Monitoring Entity	Usefulness
Pulse Pressure monitoring	Dynamic marker of threatened cardiac output, the combined effect of dynamic hyperinflation, and may result in decreasing cardiac output through decreased venous return and direct compression of the cardiac chambers.
Urine Output	Surrogate for effective tissue perfusion. Urine output is a direct marker for end-organ function and a decrease could precede the development of hemodynamic shock. Provides an early marker for intervention in most situations
Daily Cardiac Output estimation and RV function using point of care ultrasound (POCUS)	Daily assessment of RV function and estimation of cardiac output is valuable in patients on mechanical ventilation because of the complex interaction between the cardiac and pulmonary systems in response to positive pressure ventilation. Assessment of intravascular volume and the effects of dynamic hyperinflation can be readily apparent.
P_peak_	While absolute P_peak_ values are less valuable in bronchospasm, establishing a trend may be useful to assess response to therapy
auto-PEEP	Total PEEP measurement using an end-inspiratory hold manoeuvre is necessary for patients with a high risk of developing dynamic hyperinflation and provides a direct measure of auto-PEEP; this information in the context of invasive hemodynamic monitoring is a useful guide to titration of ventilatory strategies.
Airway resistance	Many current ventilators are able to measure airway resistance in addition to the pressures described above; establishing a trend of airway resistance can help assess response to therapy.
Chest radiograph	Due to the high risk of pneumothorax associated with mechanical ventilation in status asthmaticus, frequent chest radiographs may be useful. In addition, monitoring of indwelling catheters, endotracheal tubes, and ECMO cannulae may be necessary depending on the clinical context.
Lung Ultrasound	The presence of A-lines with lung sliding in the anterolateral regions without alveolar consolidations or pleural effusions in the posterolateral areas (A-profile without PLAPS) may be useful for diagnosis and monitoring for development of complications of NFA including pneumothorax, pneumonia, and pulmonary oedema [80].

ECMO: extracorporeal membrane oxygenation; NFA: near-fatal asthma; PEEP: positive end-expiratory pressure; PLAPS: posterolateral alveolar and/or pleural syndrome; POCUS: point of care ultrasound; P_peak_: peak pressure; RV: right ventricle.

## Data Availability

No new data were created or analyzed in this study. Data sharing is not applicable to this article.
